# E3 UBIQUITIN LIGASE2 SIAH2 MAY REGULATE AGE-ASSOCIATED LIPID TURNOVER IN ADIPOSE TISSUE MACROPHAGES

**DOI:** 10.1093/geroni/igae098.3654

**Published:** 2024-12-31

**Authors:** Bhaswati Ghosh

**Affiliations:** Pennington Biomedical Research Center, BATON ROUGE, Louisiana, United States

## Abstract

The global adult population with obesity is rapidly increasing. One of the aging hallmarks is accumulation of pro-inflammatory cells, which disrupts the balance between lipid storage and lipolysis leading to adipose tissue (AT) expansion and obesity-related insulin resistance. The primary inflammatory contributors in AT are adipose tissue macrophages (ATMs). As in obesity, ATM accumulation increases with aging. Notably, the ubiquitin-proteasome system, the non-lysosomal degradative machinery that maintains protein homeostasis, tightly regulates inflammation and declines with age. We hypothesized that aging downregulates proteasomal activity in ATMs resulting in dysfunctional lipid metabolism. We previously showed the E3-ubiquitin ligase Seven-in-Absentia mammalian homolog2 (Siah2) is crucial for promoting adipogenesis in pre-adipocytes and regulates obesity-mediated AT inflammation. Recently we found Siah2 is present in immune cells including macrophages. Here, we generated a macrophage-specific Siah2 knockout (SKM) mouse model to investigate the role of Siah2 in ATM lipid metabolism. We co-cultured primary SKM-bone marrow-derived macrophages (SKM-BMDMs) or wild-type-bone marrow-derived macrophages (WT-BMDMs) in presence of gonadal white adipose tissue from lean mice as a model of bone marrow-derived ATMs. Interestingly, SKM-ATMs accumulate more, and larger lipid droplets than WT-ATMs via increased lipid uptake. Furthermore, Siah2KO in ATMs suppresses lysosomal delivery of lipid droplets, and the SKM-ATM lysosomes are enlarged compared to WT-ATMs. Consistently, SKM-BMDMs are pro-inflammatory and show decreased oxygen consumption rate indicative of a switch to glycolytic metabolism. Altogether, our findings indicate Siah2 functions as a critical lipid sensor in ATMs, raising the possibly that aging downregulates Siah2 activity in ATMs leading to declined lipid turnover.

